# Timing-dependent effects of melatonin supplementation on exercise performance and exercise-induced muscle damage: a systematic review and meta-analysis

**DOI:** 10.3389/fnut.2026.1742464

**Published:** 2026-02-13

**Authors:** Jintao Guo, Lin Zhou, Jinfa Gu, Jie Sun, Guangsong Liu, Chao Wei

**Affiliations:** 1 School of Physical Education, Shandong University, Jinan, Shandong, China; 2 Beijing Sport University, Beijing, China; 3 Exercise & Sports Science Programme, School of Health Sciences, Universiti Sains Malaysia, Kubang Kerian, Kelantan, Malaysia; 4 University of Jinan, Jinan, Shandong, China

**Keywords:** athletic performance, endurance performance, meta-analysis, muscle damage biomarkers, nutritional supplementation, systematic review

## Abstract

**Background:**

Melatonin, an endogenous neurohormone with both chronobiotic and antioxidant properties, has been proposed as a nutritional aid for recovery and performance optimization. However, its timing-dependent effects on athletic performance remain unclear. This systematic review and meta-analysis aimed to evaluate the timing-dependent effects of melatonin supplementation on exercise performance and exercise-induced muscle damage in athletes and physically active individuals.

**Methods:**

A comprehensive search of PubMed, Web of Science, SPORTDiscus, and Cochrane Library was conducted from inception to September 2025. Only randomized controlled trials (RCTs) published in English examining melatonin versus placebo effects on athletic performance and muscle damage biomarkers were included.

**Results:**

In total, 19 RCTs involving 266 participants met the inclusion criteria. Meta-analysis revealed small effects on explosive power (SMD = 0.29, 95% CI: 0.05 to 0.53, *p* = 0.02, *I*^2^ = 0%) and moderate effects on endurance performance (SMD = 0.58, 95% CI: 0.29 to 0.87, *p* < 0.001, *I*^2^ = 46%). Melatonin significantly reduced creatine kinase levels (SMD = 0.59, 95% CI: 0.29 to 0.89, *p* < 0.0001, *I*^2^ = 0%), with non-significant effects on lactate dehydrogenase (SMD = 0.45, 95% CI: −0.03 to 0.94, *p* = 0.07, *I*^2^ = 56%). Subgroup analyses revealed timing-dependent effects: evening administration with >6-h exercise intervals produced superior benefits for both endurance performance and explosive power compared to daytime administration or shorter intervals. Higher doses (6–10 mg) and athlete populations demonstrated greater improvements, while adolescents (≤18 years) showed enhanced explosive power responses.

**Conclusion:**

These findings suggest that melatonin supplementation, particularly when administered in the evening with adequate timing intervals, enhances endurance performance and reduces exercise-induced muscle damage in athletes during intensive training periods.

**Systematic review registration:**

https://www.crd.york.ac.uk/PROSPERO/view/CRD420251178430, Identifier (CRD420251178430).

## 1 Introduction

Optimizing athletic performance while attenuating exercise-induced muscle damage (EIMD) is a central goal in sports science and applied training practice. High-intensity or prolonged exercise inevitably induces mechanical strain, oxidative stress, and inflammatory responses within skeletal muscle, which can manifest as delayed-onset muscle soreness, elevated muscle damage biomarkers, and transient impairments in force production and endurance capacity (1–3). When recovery is incomplete, repeated EIMD may accumulate across training microcycles, ultimately compromising training adaptation and increasing the risk of overreaching or injury (4–6).

Melatonin is an endogenous indoleamine mainly secreted by the pineal gland with a well-known role in regulating circadian rhythms and sleep–wake cycles (7, 8). Beyond its chronobiotic effects, melatonin exerts potent antioxidant and anti-inflammatory actions, acting both as a direct scavenger of reactive oxygen and nitrogen species and as a modulator of antioxidant defense systems (9–12). Experimental and clinical studies suggest that melatonin can reduce oxidative stress, limit secondary tissue damage, and modulate immune responses after various noxious stimuli (13–16). These properties make melatonin a theoretically attractive candidate for protecting skeletal muscle from exercise-induced damage and for supporting recovery in athletes and physically active individuals.

At the same time, melatonin’s hypnotic and sedative properties raise important concerns for its use around exercise. Daytime or pre-exercise ingestion may induce drowsiness, slow reaction time, and negatively affect vigilance or neuromuscular performance, especially in tasks requiring high levels of arousal and coordination (17–19). Moreover, melatonin secretion and responsiveness are strongly time-dependent, being closely linked to the light–dark cycle and circadian phase (8, 20). As a result, the net impact of melatonin supplementation on athletic performance and EIMD is expected to depend critically on the timing of ingestion (daytime vs. evening, proximity to exercise) and the duration of supplementation (acute vs. repeated dosing).

Over the past decade, randomized controlled trials have investigated the effects of melatonin supplementation on various aspects of athletic performance (e.g., endurance tests, sprinting, strength, and power) and on blood biomarkers of muscle damage and tissue stress [e.g., creatine kinase (CK), lactate dehydrogenase (LDH)] in athletes and physically active individuals. However, findings are inconsistent: some studies report attenuated rises in muscle damage biomarkers or improved recovery, whereas others show trivial or even potentially detrimental effects on performance outcomes (5, 13, 16, 21–23). Furthermore, most narrative reviews and previous quantitative syntheses have focused on the general antioxidant or clinical effects of melatonin, with limited emphasis on sports-specific endpoints. To the best of our knowledge, no systematic review has comprehensively quantified the impact of melatonin on both athletic performance and EIMD while explicitly considering supplementation timing and duration as potential effect modifiers.

Therefore, this systematic review and meta-analysis aimed to synthesize randomized controlled trials evaluating melatonin supplementation in athletes and physically active individuals, with two primary objectives: (1) to quantify the effects of melatonin, compared with placebo, on athletic performance, with particular focus on endurance and explosive power outcomes; and (2) to determine whether melatonin attenuates exercise-induced muscle damage, primarily indexed by serum CK and LDH. A secondary objective was to explore whether the effects of melatonin on these outcomes are modified by supplementation timing (time of day and administration-to-exercise interval) and supplementation duration (acute vs. short-term protocols). By clarifying these questions, this review seeks to provide evidence-based guidance on whether, when, and how melatonin supplementation may be beneficial or detrimental in athletic settings.

## 2 Materials and methods

This systematic review and meta-analysis was conducted following the Preferred Reporting Items for Systematic Reviews and Meta-Analyses (PRISMA) guidelines (1) and was registered (2) in PROSPERO (registration number: CRD420251178430), an international prospective register of systematic reviews.

### 2.1 Literature search

Two authors (JG and XY) independently conducted a comprehensive literature search in four electronic databases—PubMed, Web of Science (WoS), SPORTDiscus (via EBSCOhost), and the Cochrane Library—to identify studies published from inception to September 2025. Boolean logic operators (“AND,” “OR”) were applied to combine relevant keywords related to melatonin supplementation, exercise performance, and muscle damage biomarkers.

Reference lists of included studies were also manually screened to identify any additional relevant publications. The search was restricted to English-language articles. Any disagreements between the two reviewers (JG and XY) were resolved through discussion, and if consensus could not be reached, a third reviewer (LP) adjudicated. The complete search strategy for each database is summarized in Supplementary Table S1.

### 2.2 Literature search inclusion and exclusion criteria

Inclusion criteria were based on the PICOS framework: (1) population: athletes or physically active individuals of all training levels (recreational, amateur, professional, and elite), with no restrictions on age or sex; (2) intervention: melatonin supplementation as either a standalone intervention or combined with other treatment modalities, with no restrictions on melatonin dosage, timing of administration, or mode of administration; (3) comparison: control groups receiving placebo combined with regular exercise training or testing protocols, with control groups undergoing identical exercise protocols as intervention groups, with only the supplement being replaced by placebo instead of melatonin; (4) outcome: studies reporting primary quantitative data for evaluating exercise performance outcomes (including but not limited to aerobic capacity, anaerobic performance, muscle strength, power output, endurance, and reaction time) and/or exercise-induced injury markers (including but not limited to oxidative stress markers, inflammatory markers, muscle damage markers, and immune function indicators), with sufficient statistical parameters (means, standard deviations, and sample sizes) for meta-analysis; (5) study design: randomized controlled trial (RCT) or randomized crossover trial designs; (6) peer-reviewed published articles.

### 2.3 Data extraction

The primary data for this study were outcome measures of exercise performance-related indicators and exercise-induced muscle damage markers assessed in the included studies. Other relevant data extracted included study reference, study design, participant characteristics (sample size, age, gender distribution, training status, training experience, and physical fitness level), intervention details (melatonin dose, timing of administration, administration method, and supplementation duration), control conditions (placebo type and administration method), outcome assessment methods, and primary study results (statistical parameters such as means, standard deviations, and sample sizes).

For studies where full-text articles were unavailable or outcome data were not directly reported in sufficient detail for meta-analysis, the corresponding authors were contacted via email to request the missing information. Authors were given a 14-day response period. Studies for which no response was received within this timeframe or for which requested data could not be obtained were excluded from the quantitative synthesis.

Only studies that provided directly reported mean values and standard deviations (SD) were included in the meta-analysis. For studies reporting standard error (SE) instead of SD, data were converted using the following formula:


SD=SE×√n

where *n* represents the sample size of the respective group. This conversion was performed to ensure consistency in effect size calculations across all included studies. All data conversions were independently verified by two reviewers to ensure accuracy.

Exercise performance outcomes included various field and laboratory tests assessing aerobic capacity, anaerobic performance, muscle strength, power output, endurance, and reaction time. Biochemical markers of muscle damage, oxidative stress, and inflammation were assessed through venous blood sampling in all included studies. Blood samples were collected at baseline and at predetermined time points post-exercise according to each study’s protocol, and analyzed using standard laboratory procedures. The specific categorization and operational definitions of performance domains and biomarker classifications are detailed in Sections 2.5.1 and 2.5.2.

### 2.4 Risk of bias assessment and summary of evidence

The included studies were assessed for risk of bias using the Cochrane risk-of-bias tool for randomized trials—version 2 (ROB2) tool (3). Five domains are included in the ROB2 as bias arising from the randomization process, bias due to deviations from intended interventions, bias due to missing outcome data, bias in measurement of the outcome, and bias in selection of the reported result. Two reviewers independently assessed each included study, and any disagreements were resolved through discussion or consultation with other reviewers. The risk-of-bias judgment for the RCTs was considered as low risk of bias, some concerns, or high risk of bias in each domain.

After conducting the meta-analysis, the quality of evidence for each outcome was further assessed using the Grades of Recommendation, Assessment, Development, and Evaluation (GRADE) framework. The justifications of GRADE are based on potential study limitations, the consistency of effects, the precision of results, and the relevance (directness) of evidence, providing a comprehensive appraisal of the pooled evidence’s quality. According to these domains, the GRADE system can justify the certainty or confidence in the evidence as a continuum of four categories, ranging from high, moderate, low, and very low (4).

### 2.5 Statistical analysis

Data extraction and statistical analyses were performed independently by two researchers. The standardized mean difference (SMD; Cohen’s d) with corresponding 95% confidence intervals (CI) was chosen as the primary effect size to evaluate the effects of melatonin supplementation versus placebo on exercise performance and muscle damage biomarkers.

#### 2.5.1 Performance domain categorization

Exercise performance outcomes were systematically categorized into four distinct domains based on the primary physiological systems and motor abilities assessed: (1) explosive power performance—assessments of anaerobic power output and rapid force generation, including power output measurements, squat jump (SJ), and countermovement jump (CMJ) tests; (2) endurance performance—tests measuring aerobic capacity and sustained exercise tolerance, comprising long-distance time-to-completion protocols (e.g., 4 km cycling time trial and 32.2 km cycling time trial) and incremental exhaustion tests; (3) speed performance—assessments of rapid movement execution and neural processing speed, including reaction time measurements and short-distance sprint tests (e.g., 10-m, 20-m, and 30-m sprint times); (4) strength performance—maximal voluntary force production measures, comprising handgrip dynamometry, bench press capacity, and squat strength tests.

This classification framework was established *a priori* based on standard exercise physiology taxonomies and sport science assessment protocols. When studies reported multiple outcomes within a single domain, the most representative or frequently reported measure was selected for primary analysis to avoid outcome redundancy. Classification decisions were independently reviewed by two authors (JG and WS), with any ambiguities resolved through consensus discussion with a third reviewer (WC). This systematic categorization enabled domain-specific subgroup analyses to identify differential effects of melatonin across distinct performance dimensions.

#### 2.5.2 Muscle damage biomarker categorization

All biochemical markers were obtained through venous blood sampling and analyzed using standard enzymatic or colorimetric laboratory assays. Exercise-induced muscle damage and recovery status were evaluated through three categories of biochemical markers: (1) muscle damage indicators—direct markers of sarcomeric disruption and membrane permeability, including creatine kinase (CK), lactate dehydrogenase (LDH), aspartate aminotransferase (ASAT), and alanine aminotransferase (ALAT). This biomarker stratification was based on established exercise biochemistry literature and enabled a comprehensive assessment of melatonin’s effects on multiple pathophysiological pathways involved in exercise-induced tissue stress and recovery.

To maintain consistency in interpretation across all outcomes, effect sizes were coded such that positive SMDs uniformly indicate beneficial effects of melatonin supplementation. For performance outcomes (endurance, explosive power, strength, and speed), positive SMDs represent improved performance in the melatonin group. For muscle damage biomarkers (CK, LDH, ASAT, and ALAT), where lower values indicate less damage and thus better outcomes, the direction was reversed by multiplying raw SMDs by −1 before pooling. Therefore, positive SMDs for biomarkers represent lower concentrations (reduced damage) in the melatonin group compared to placebo, consistent with a protective effect. This approach ensures that across all analyses, positive effect sizes indicate benefits favoring melatonin supplementation.

#### 2.5.3 Subgroup, sensitivity, and publication bias analyses

Meta-analyses were conducted using Review Manager software (RevMan, version 5.4; Cochrane Collaboration, London, UK) based on a random-effects model (DerSimonian–Laird method) to account for potential between-study heterogeneity. Statistical heterogeneity was assessed using the *I*^2^ statistic, with *I*^2^ < 25% indicating low heterogeneity, 25–50% moderate heterogeneity, and >50% substantial heterogeneity. When *I*^2^ exceeded 50%, sources of heterogeneity were further explored through subgroup and sensitivity analyses.

When heterogeneity was low or moderate (*I*^2^ ≤ 50%), fixed-effect models were applied; when substantial heterogeneity was present (*I*^2^ > 50%), random-effects models were used.

To facilitate interpretation of between-study variability, we pre-specified a set of subgroup categories that were applied across both performance outcomes and muscle-damage biomarkers. Among all extracted outcomes, explosive power, endurance performance, creatine kinase (CK), and lactate dehydrogenase (LDH) were considered the primary outcomes for more in-depth analyses.

Subgroup analyses were stratified by the following predefined variables: population type (athletes vs. non-athletes); age (≤18 years vs. >18 years);

Time of melatonin administration (daytime vs. evening); administration-to-exercise interval (≤ 2 h vs. 2–6 h vs. > 6 h); dosage (3 mg vs. 5 mg vs. 6 mg vs. 8–10 mg); supplementation duration (acute vs. short-term multi-day intake); performance index type (endurance, explosive power, muscular strength, neuromotor coordination, and speed performance).

For studies including multiple intervention arms, each eligible comparison was treated as an independent dataset, with shared control groups adjusted to avoid double-counting. Sensitivity analysis was performed using the leave-one-out method to assess the influence of each study on pooled estimates.

Publication bias was assessed by visual inspection of funnel plots and Egger’s regression test (*t*-test), both conducted using Stata software (version 17.0; StataCorp, College Station, TX, USA). When bias was detected, the trim-and-fill method was applied to estimate the bias-corrected effect size. A two-tailed *p* < 0.05 was considered statistically significant.

We chose categorical subgroup analysis rather than meta-regression for the following reasons: (1) insufficient sample sizes for meta-regression (*n* = 6–12 studies per outcome; meta-regression requires ≥10 studies per covariate); (2) moderator variables showed discrete clustering rather than continuous distribution (e.g., most studies used <2 h, 2–6 h, or >6 h intervals, with few intermediate values); (3) meta-regression with multiple covariates and interactions would risk overfitting and spurious findings given limited degrees of freedom; and (4) categorical subgroups provide more clinically interpretable and actionable guidance. All subgroup categories were pre-specified in our PROSPERO protocol to minimize Type I error risk from data-driven analysis.

## 3 Results

### 3.1 Study selection

**Figure 1:**
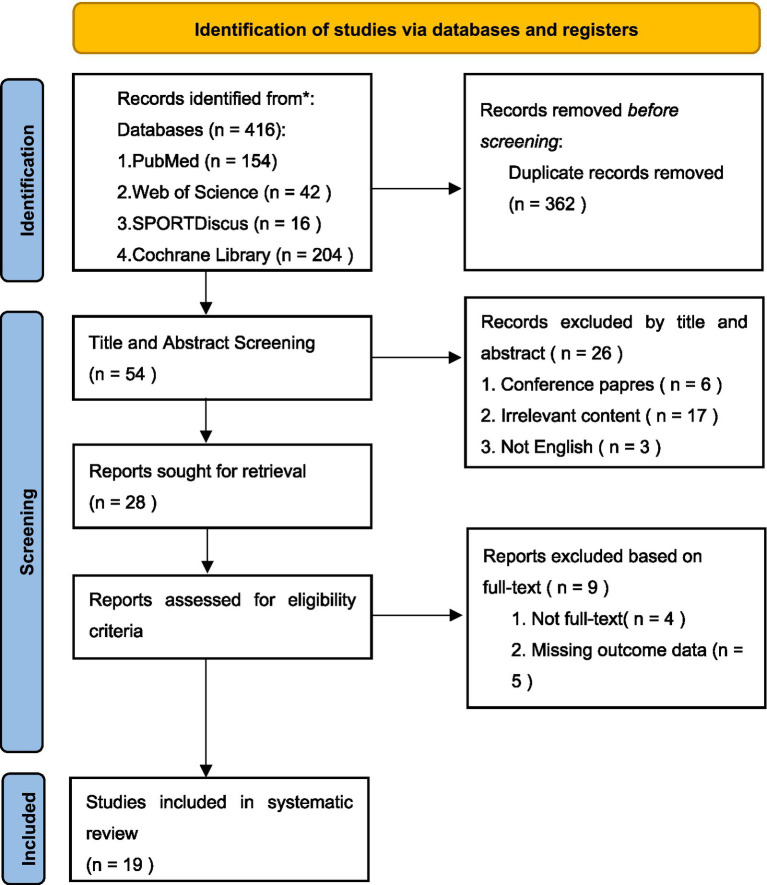
PRISMA.

Figure 1 presents the flowchart of the literature screening process. A total of 416 relevant records were identified through searches of four databases (PubMed: 154 articles, Web of Science: 42 articles, SPORTDiscus: 16 articles, Cochrane Library: 204 articles). After removing 362 duplicate publications, 54 articles proceeded to the screening process. During the title and abstract screening phase, 26 articles were excluded. During the full-text screening phase, 9 articles were excluded due to unavailable full text (*n* = 5) and missing outcome measures (*n* = 4). Finally, 19 studies were included in the meta-analysis (5–23).

### 3.2 Characteristics of included studies

This systematic review and meta-analysis ultimately included 19 randomized controlled trials involving 266 participants. The publication years ranged from 2005 to 2025, with sample sizes ranging from 10 to 30 participants.

Regarding participant characteristics, five studies focused on adolescent athletes (≤18 years, mean age ~16–17 years) (9–15, 17), while 14 studies involved adults (>18 years, age range 20–35 years) (5–8, 16, 18–23). In total, 13 studies recruited competitive or professional athletes (primarily soccer and volleyball players), while six studies involved physically active non-athletes or recreationally trained individuals.

Concerning intervention design, melatonin dosage ranged from 3 mg to 10 mg, with 3 mg (7), 5 mg (5, 8, 11, 13, 15–17), 6 mg (6, 12, 14, 20–23), and 8–10 mg groups (9, 10, 16, 17, 19). Nine studies implemented evening/nocturnal ingestion (19:00–22:00 or before sleep) (24–30), eight used daytime administration (morning or 15–60 min pre-exercise) (28–31, 36–38, 44, 46), and two used mid-day ingestion. A total of 14 studies employed acute protocols, while 5 used short-term multi-day interventions (6 days or 4 weeks).

Performance outcomes included endurance capacity (time to exhaustion and cycling time trials) (28–31, 45), explosive power (Wingate test and vertical jump), muscular strength (handgrip and 1RM) (28, 38), neuromotor coordination (repeated sprint ability), and speed performance (24, 26, 28, 36, 40, 41). Biomarkers assessed comprised muscle damage indicators (CK, LDH, ASAT, ALAT, and CRP in 13 studies) (24–27, 29, 30, 32, 33, 35–37, 42, 43, 45).

Regarding safety, 16 studies reported no adverse events (28–38, 41–43, 45, 46), while 3 documented melatonin-induced drowsiness or reduced alertness with daytime administration (38, 40, 41), suggesting timing is critical to avoid performance-impairing sedative effects (see Table 1).

**Table 1:** Characteristics of included studies.

Author (year)	Participant characteristics	Experimental group (melatonin)	Supplementation duration	Exercise intervention	Main outcome—exercise performance	Main outcome—tissue damage/biomarkers
Atkinson et al. (31) (2005)	Physically active adults (non-athletes)	5 mg melatonin orally, mid-day ingestion; tests at 13:00 and 17:00	Acute effect	4-km cycling time trial	No significant effect on completion time, power output, or RPE	Not reported
Brandenberger et al. (34) (2018)	Endurance-trained male cyclists (VO₂max > 52 mL kg^−1^ min^−1^)	5 mg melatonin 15 min pre-exercise 32.2-km cycling TT	Acute effect	32.2-km cycling time trial	No significant difference in time, power, HR, or RPE between trials	Not reported
Beck et al. (32) (2018)	Moderately active men (non-athletes)	6 mg melatonin 30 min pre-exercise	Acute effect	Time to exhaustion at individual maximal aerobic capacity (iMAC)	Increased endurance time ↑	No significant difference in CK, LDH
Cheikh et al. (24) (2020)	Adolescent competitive volleyball athletes	10 mg melatonin at 22:00 after evening exhaustive exercise; tested the next morning	Acute effect	RAST sprint test (evening and next morning)	Improved next-morning performance: ↑ Ppeak, ↑ Pmean, ↓ fatigue index, ↓ total time	↓ CK, LDH, ASAT, us-CRP, MDA, and homocysteine
Ben Dhia et al. (33) (2022)	Overweight/obese adults (non-athletes)	3 mg melatonin 40 min pre-exercise	Acute effect	High-intensity interval exercise (8 × 1 min @90% MAP/2 min @45% MAP)	No direct performance variable (only HR and metabolic data)	↓ CK, ASAT, ALAT, CRP, WBC, MDA, AOPP
Cheikh et al. (25) (2018)	Adolescent elite volleyball players	3 mg melatonin at 22:00 for 4 weeks	Acute effect	Nighttime sleep monitoring + standard training	Improved sleep quality and training readiness; no fatigue increase	↓ MDA, ↓ CK, ↑ TAS—melatonin enhanced antioxidant status and reduced muscle damage markers
Farjallah et al. (27) (2018)	Professional soccer players	5 mg melatonin daily at 19:00 for 6 days during the intensive training camp	Acute effect	6-day training camp + RSA (6 × 40 m sprints)	Attenuated post-camp performance decline – better first and second sprints vs. placebo	↓ MDA, ↓ CK, ↓ LDH, ↑ SOD; less muscle pain and oxidative stress
Ghattassi et al. (28) (2024)	Professional soccer players	6 mg melatonin 30 min pre-exercise; morning exercise for 7 days	6 days	Progressive maximal aerobic test (Vameval + RSA)	No change in max speed or time to exhaustion	↓ MDA, ↓ CK, ↓ LDH, ↑ SOD, and GPx; melatonin protected against oxidative stress
Farjallah et al. (35) (2020)	Elite soccer players	6 mg melatonin for 7 days, evening intake	6 days	Intensive soccer training microcycle + repeated sprint and RSA tests	Slight improvement in RSA recovery; faster performance restoration	↓ CK, ↓ LDH, ↓ MDA, ↑ SOD, ↑ CAT; reduced muscle damage and oxidative stress
Farjallah et al. (36) (2022)	Professional soccer players	6 mg melatonin 30 min pre-exercise; maximal running exercise	Acute effect	Running to exhaustion at 100% MAS	No effect on time to exhaustion or RPE	↓ MDA, ↓ CK, ↓ LDH, ↓ GPx change, ↓ UA increase, ↑ antioxidant status → cellular protection
Ghattassi et al. (40) (2014)	Professional soccer players	8 mg melatonin 30 min pre-exercise	Acute effect	Morning Wingate 30-s anaerobic test	↓ Peak and mean power; ↑ fatigue index; reduced alertness	No biochemical markers assessed; performance decline linked to sleepiness
Ghattassi et al. (41) (2016)	Professional soccer players	8 mg of melatonin taken in the morning	Acute effect	Short-term maximal tests at morning, at noon, and in the afternoon	↓ Performance only in the morning; no effect later in the day	Not reported
Farjallah et al. (37) (2022)	Professional soccer players	6 mg melatonin 30 min pre-exercise	6 days	Running to exhaustion at 100% MAS	No significant change in time to exhaustion	↓ CK, ↓ LDH, ↓ MDA, ↑ SOD, and CAT—reduced muscle damage and oxidative stress
Ghattassi et al. (28) (2024)	Professional soccer players	8 mg melatonin before sleep (nocturnal ingestion)	Acute effect	Next-morning battery (HG, SJ, MAT, Wingate)	↑ Handgrip and jump height, ↑ P_peak_ and P_mean_ power, ↓ fatigue index, and RPE	No differences in [La] or [GL]; improved subjective recovery
Trionfante et al. (39) (2017)	Healthy, physically active college students	6 mg melatonin 30 min pre-exercise	Acute effect	30-min graded treadmill exercise (Naughton protocol)	Earlier crossover point (CHO > 50% energy); ↑ carbohydrate utilization	No tissue-damage markers assessed; melatonin shifted substrate use toward CHO
Mero et al. (38) (2006)	Resistance-trained men, healthy, age ≈ 24 y	6 mg melatonin 60 min pre-exercise; heavy resistance exercise (daytime)	Acute effect	80-min hypertrophic resistance session (bench press, squat, leg curl, etc.)	No effect on jump height, 1RM squat, or bench press; GH AUC ↓ under melatonin vs. placebo	↓ GH AUC, no change in cortisol/testosterone; no oxidative or damage biomarkers measured
Leonardo-Mendonça et al. (42) (2017)	Healthy, physically active adults (not athletes)	10 mg melatonin 1 h before an exhaustive treadmill run	4 weeks	Incremental treadmill test to exhaustion	↑ Time to exhaustion, ↓ HR, and RPE during exercise	↓ CK, ↓ MDA, ↓ TBARS, ↑ SOD, and CAT → reduced oxidative stress and muscle injury
Paryab et al. (30) (2021)	Male endurance-trained athletes	6 mg melatonin daily for 14 days	Acute effect	14-day endurance training program + post-test 5 km run	↑ VO₂max, ↑ running time, ↓ fatigue index	↓ CK, ↓ LDH, ↓ MDA, ↑ GPx, ↑ TAC—improved antioxidant status and recovery
Mahdi et al. (29) (2025)	Professional soccer players	5 mg melatonin for 7 nights during a congested training week	Acute effect	7-day intensive soccer training (2 sessions/day)	Maintained sprint & jump performance; less performance decrement	↓ CK, ↓ LDH, ↓ CRP, ↓ MDA, ↑ SOD, ↑ GPx; significantly attenuated oxidative and inflammatory stress

CK, creatine kinase; LDH, lactate dehydrogenase; ASAT, aspartate aminotransferase; ALAT, alanine aminotransferase; MDA, malondialdehyde; CRP, C-reactive protein; RAST, running-based anaerobic sprint test; RPE, rating of perceived exertion; MAP, maximal aerobic power; GPx, glutathione peroxidase; SOD, superoxide dismutase; CAT, catalase; TAS, total antioxidant status; iMAC, individual maximal aerobic capacity.

### 3.3 Quality assessment of included studies

**Figure 2:**
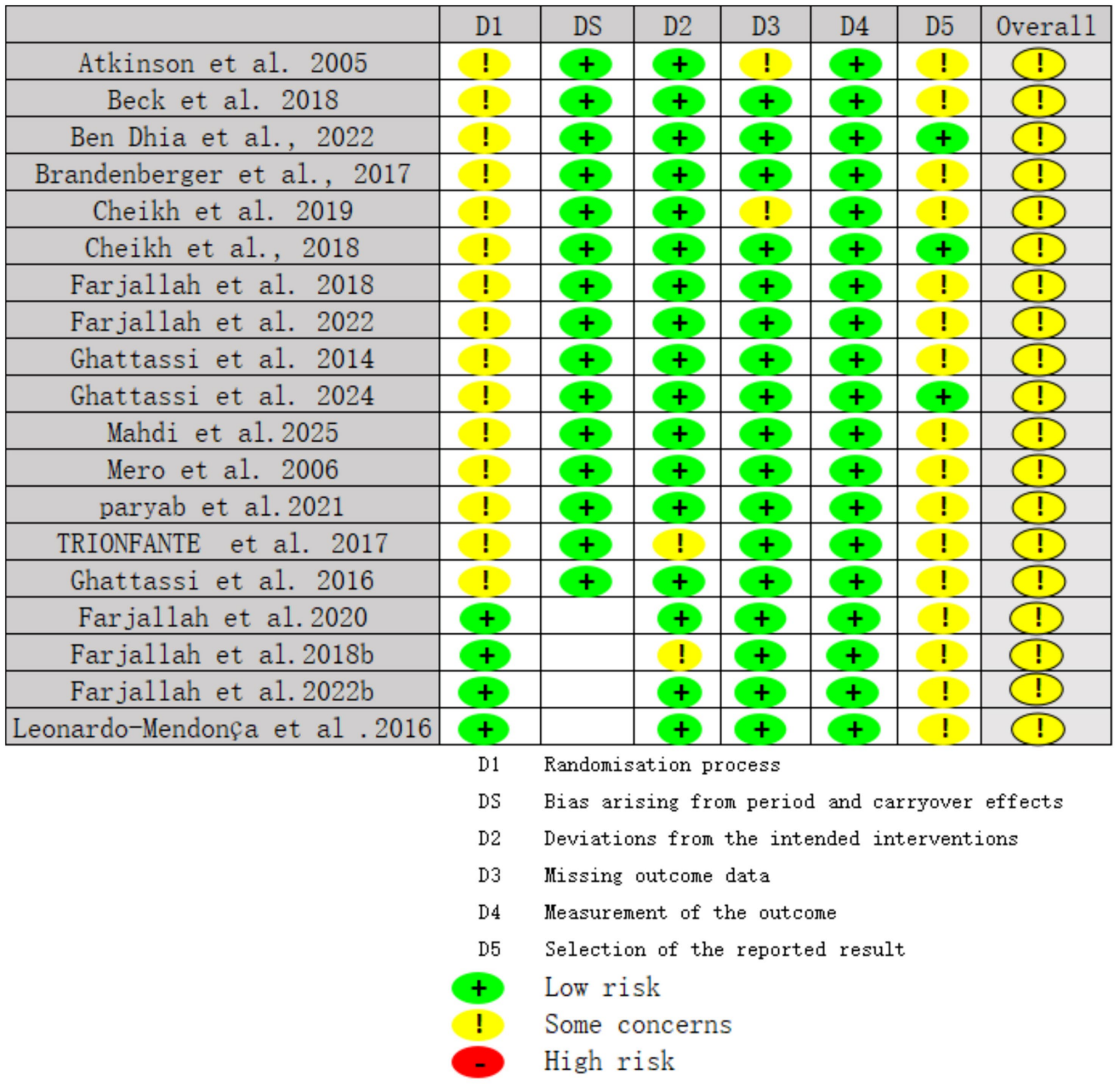
Risk of bias assessment.

**Figure 3:**
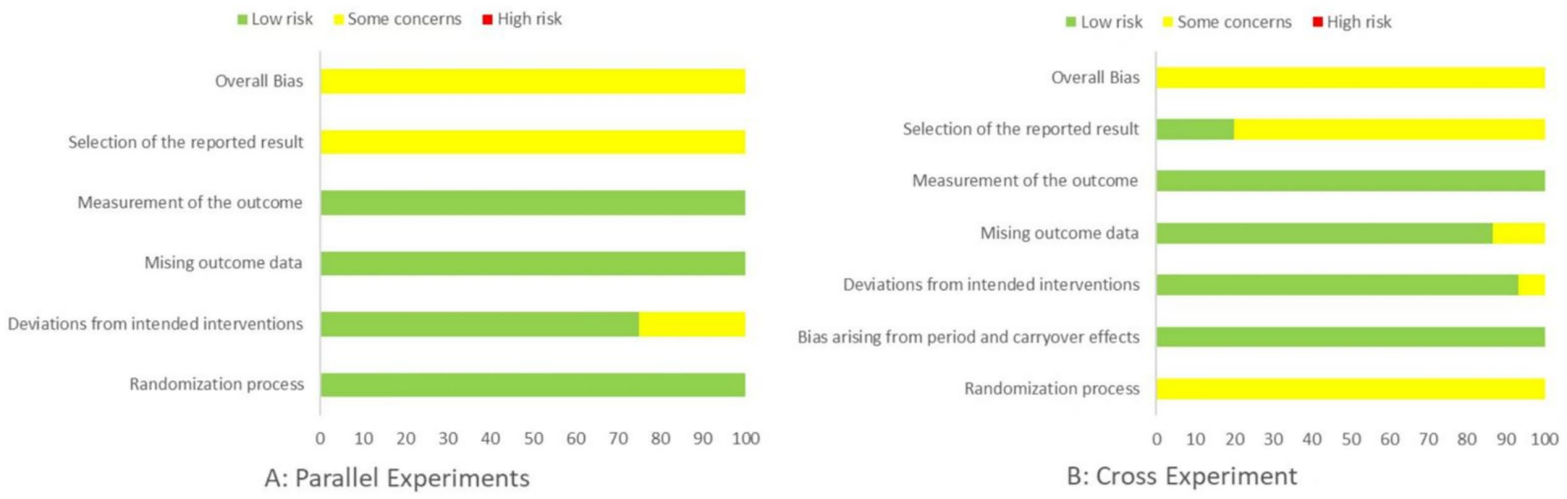
Risk of bias summary.

This systematic review assessed the risk of bias of all included randomized controlled trials using the Cochrane RoB 2.0 tool, focusing on both performance and biomarker outcomes. Among parallel-group RCTs, most were judged as having low risk of bias or some concerns, with only a minority rated as high risk (Figures 2, 3). For randomized crossover trials, in addition to RoB 2.0, we applied the DS quality checklist, and all crossover studies were rated as having low-to-moderate risk of bias and acceptable methodological quality.

### 3.4 Athletic performance meta-analysis

#### 3.4.1 Effects of melatonin on exercise performance

**Figure 4:**
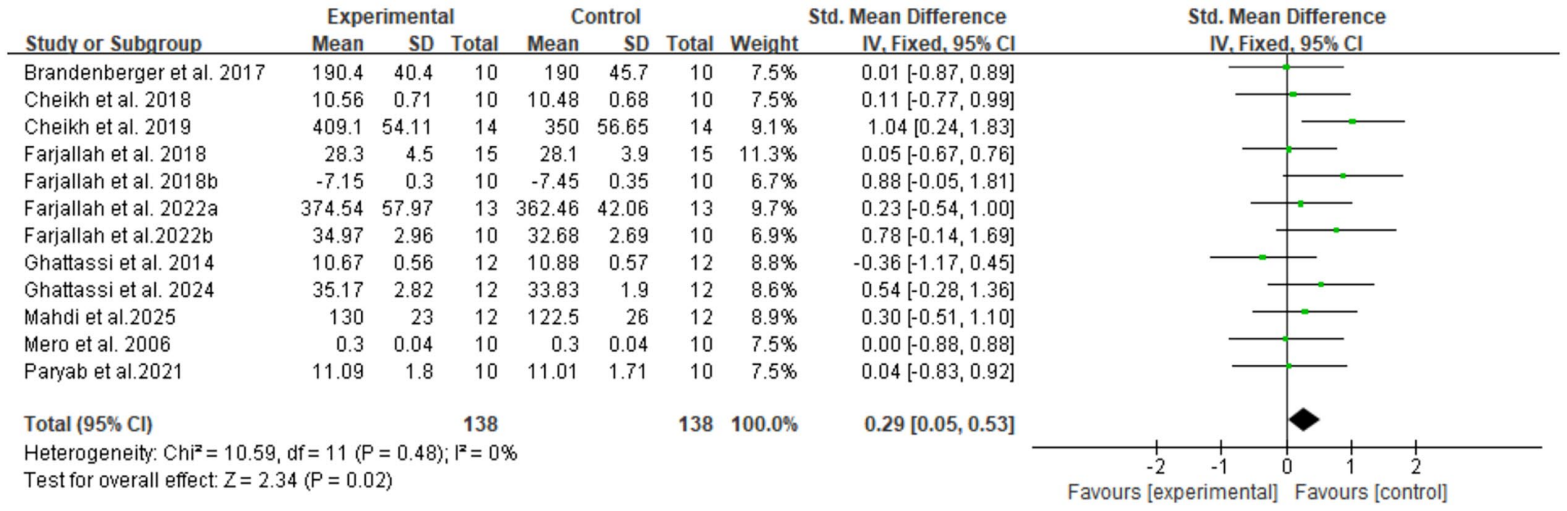
Forest plot of melatonin supplementation on explosive power.

**Figure 5:**
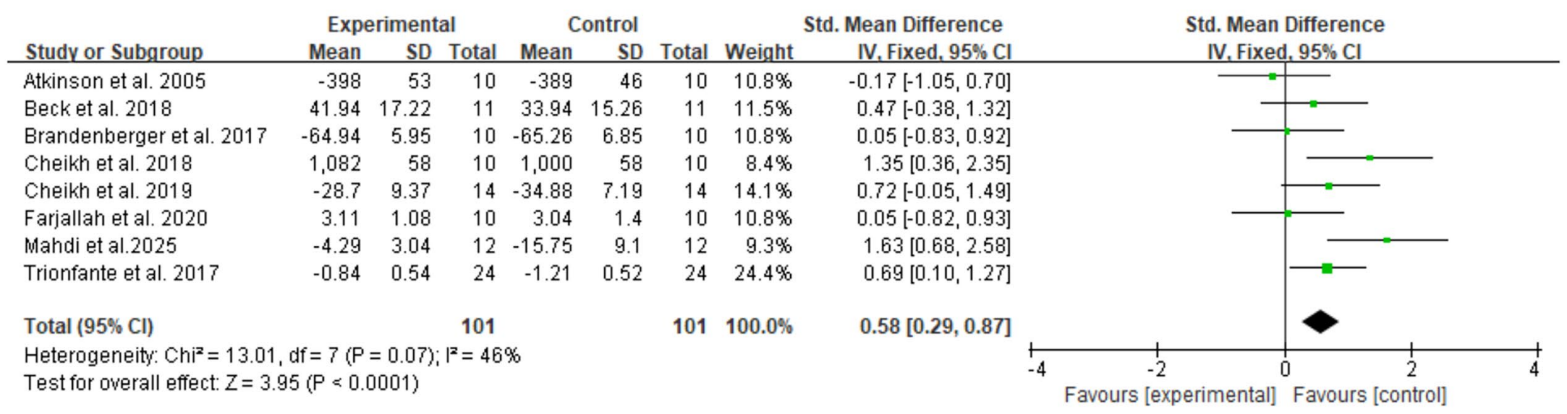
Forest plot of melatonin supplementation on endurance performance.

**Figure 6:**
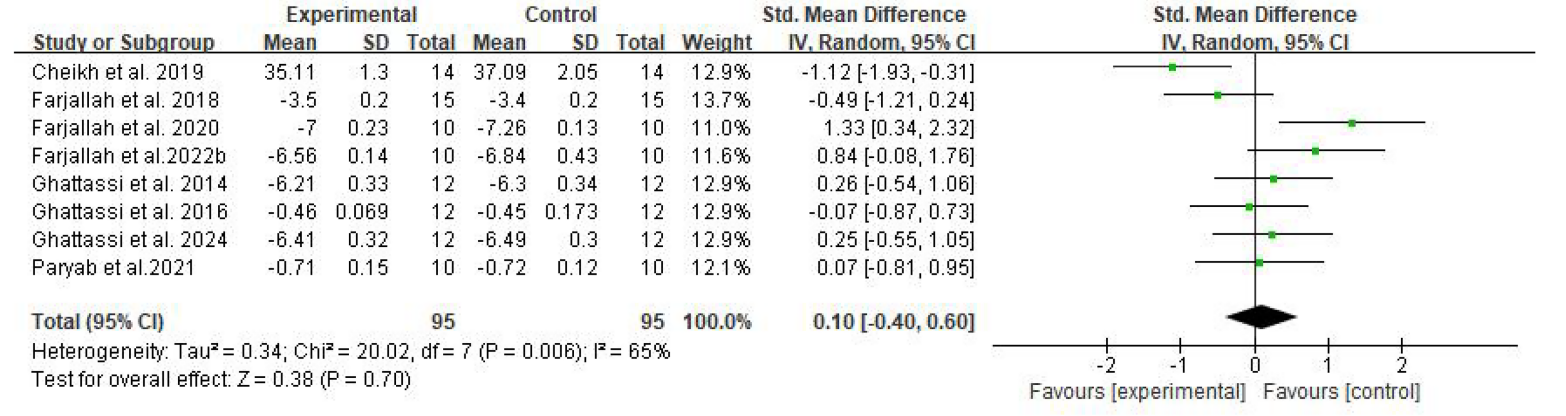
Forest plot of melatonin supplementation on speed performance.

**Figure 7:**
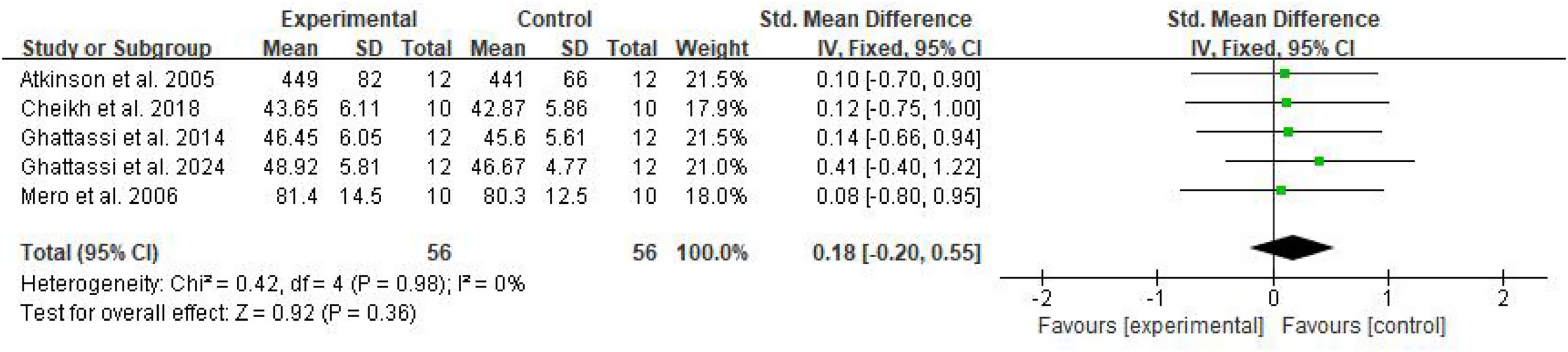
Forest plot of melatonin supplementation on muscular strength.

In total, four domain-specific meta-analyses were performed for explosive power, endurance, speed, and muscular strength outcomes. Fixed-effect models were used when between-study heterogeneity was low or moderate (*I*^2^ ≤ 50%), whereas random-effects models were applied when substantial heterogeneity was present (*I*^2^ > 50%). Standardized mean differences (SMDs) with 95% CIs were calculated, and heterogeneity was quantified using the *I*^2^ statistic (Figures 4–7).

For explosive power, melatonin produced only small effects (SMD 0.29, 95% CI: 0.05–0.53), with high-certainty evidence. The clinical meaningfulness of this small effect size is uncertain and may not translate to meaningful performance improvements in competitive settings, with a pooled SMD of 0.29 (95% CI: 0.05–0.53, *p* = 0.02, *I*^2^ = 0%), under a fixed-effect model. For endurance performance, eight trials (*n* = 100) showed a moderate beneficial effect of melatonin versus placebo (SMD 0.58, 95% CI: 0.29–0.87, *p* < 0.001, *I*^2^ = 46%), for which a random-effects model was adopted to account for between-study variability.

In contrast, melatonin did not meaningfully alter speed performance or muscular strength. The pooled analysis of eight comparisons for speed showed a trivial, non-significant effect (SMD 0.10, 95% CI: −0.40 to 0.60, *p* = 0.70, *I*^2^ = 65%) using a random-effects model due to substantial heterogeneity. Similarly, five comparisons for muscular strength yielded a small, non-significant effect (SMD 0.18, 95% CI: −0.20 to 0.55, *p* = 0.36, *I*^2^ = 0%) under a fixed-effect model, indicating that melatonin supplementation did not produce consistent improvements in maximal strength outcomes.

#### 3.4.2 Sensitivity and subgroup analyses

Sensitivity analyses using a leave-one-out approach for endurance performance and speed performance are presented in Supplementary Figure S1. Omission of any single study did not alter the pooled effect estimates or their significance. Sensitivity analyses were not conducted for explosive power and muscular strength due to *I*^2^ = 0%.

For explosive power (Table 2), adolescent participants (≤18 years) showed SMD 0.47 (95% CI: 0.14 to 0.81, *p* = 0.006, I^2^ = 9%) compared to adults SMD 0.09 (95% CI: −0.25 to 0.44, *p* = 0.6, *I*^2^ = 0%). Evening administration produced SMD 0.45 (95% CI: 0.13–0.77, *p* = 0.006, *I*^2^ = 25%), whereas daytime administration showed SMD 0.07 (95% CI: −0.29 to 0.44, *p* = 0.7, *I*^2^ = 0%). Administration-to-exercise intervals exceeding 6 h yielded SMD 0.60 (95% CI: 0.26 to 0.95, *p* = 0.0007, *I*^2^ = 0%), whereas intervals of 2 h or less showed SMD 0 (95% CI: −0.33 to 0.33, *p* = 1, *I*^2^ = 0%). Multi-day supplementation protocols produced SMD 0.83 (95% CI: 0.19–1.48, *p* = 0.01, *I*^2^ = 0%) compared to acute administration SMD 0.20 (95% CI: −0.06 to 0.46, *p* = 0.12, *I*^2^ = 0%).

**Table 2:** The subgroup analysis results of explosive power.

Study or subgroup	*n*	SMD	95% CI	*I*^2^ (%)	*p*
Age					
≤18	6	0.47	[0.14, 0.81]	9	0.006
>18	6	0.09	[−0.25, 0.44]	0	0.6
Time of melatonin administration					
Daytime	5	0.07	[−0.29, 0.44]	0	0.7
Evening	7	0.45	[0.13, 0.77]	25	0.006
Administration-to-exercise interval					
≤2 h	6	0	[−0.33, 0.33]	0	1
>6 h	6	0.6	[0.26, 0.95]	0	0.0007
Dosage					
5 mg	3	0.54	[0.01, 1.06]	8	0.04
5–10 mg	9	0.22	[−0.05, 0.49]	0	0.11
Taking cycle					
Acute effect	10	0.2	[−0.06, 0.46]	0	0.12
6 days	2	0.83	[0.19, 1.48]	0	0.01

For endurance performance (Table 3), athlete populations showed SMD 0.65 (95% CI: 0.26–1.04, *p* = 0.001, *I*^2^ = 44%) and non-athlete populations SMD 0.44 (95% CI: 0.01–0.86, *p* = 0.04, *I*^2^ = 22%). Evening administration produced SMD 0.73 (95% CI: 0.34–1.12, *p* = 0.0002, *I*^2^ = 26%) compared to daytime administration SMD 0.33 (95% CI: −0.09 to 0.76, *p* = 0.12, *I*^2^ = 35%). Administration-to-exercise intervals exceeding 6 h showed SMD 0.80 (95% CI: 0.37–1.24, *p* = 0.0003, *I*^2^ = 39%), whereas intervals ≤2 h showed SMD 0.36 (95% CI: −0.02 to 0.74, *p* = 0.06, *I*^2^ = 6%). Higher doses (5–10 mg) demonstrated SMD 0.82 (95% CI: 0.48–1.17, *p* < 0.00001, *I*^2^ = 0%), while 5 mg doses showed SMD −0.02 (95% CI: −0.53 to 0.48, *p* = 0.93, *I*^2^ = 0%).

**Table 3:** The subgroup analysis results of endurance performance.

Study or subgroup	*n*	SMD	95% CI	*I*^2^ (%)	*p*
Population					
Athlete	5	0.65	[0.26, 1.04]	44	0.001
No-athlete	3	0.44	[0.01, 0.86]	22	0.04
Age					
≤18	3	0.66	[0.16, 1.16]	46	0.009
>18	5	0.5	[0.15, 0.85]	36	0.005
Time of melatonin administration					
Daytime	3	0.33	[−0.09, 0.76]	35	0.12
Evening	5	0.73	[0.34, 1.12]	26	0.0002
Administration-to-exercise interval					
≤2 h	4	0.36	[−0.02, 0.74]	6	0.06
>6 h	4	0.8	[0.37, 1.24]	39	0.0003
Dosage					
5 mg	3	−0.02	[−0.53, 0.48]	0	0.93
5–10 mg	5	0.82	[0.48, 1.17]	0	<0.00001
Time-to-exhaustion vs. time-trial					
Time-to-exhaustion	4	0.62	[0.16, 1.08]	17	0.008
Time-trial	3	0.38	[−0.51, 1.28]	65	0.4

SMD, standardized mean difference; CI, confidence interval.

#### 3.4.3 Publication bias

**Figure 8:**
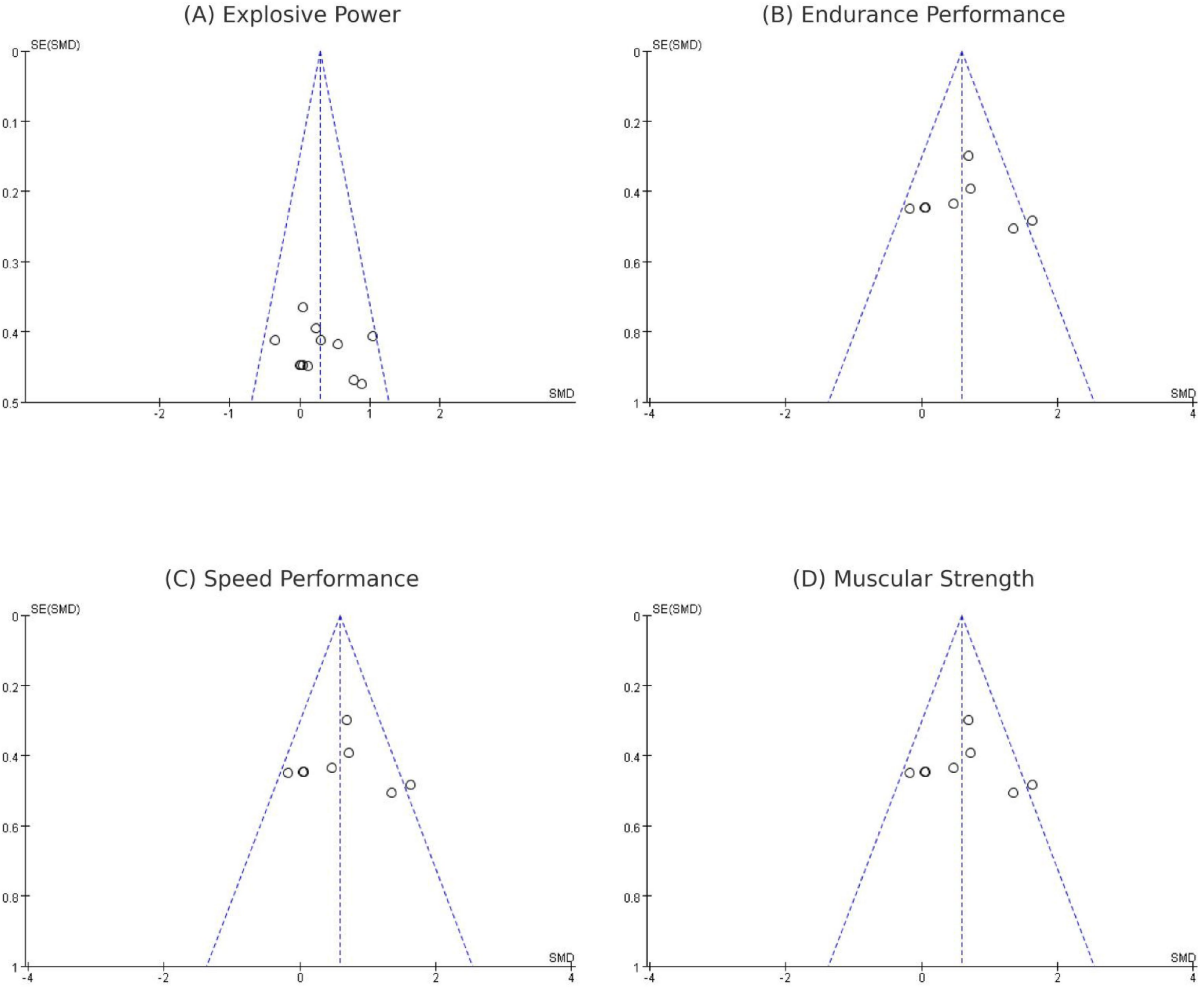
Funnel plot of melatonin supplementation on athletic performance as **(A-D)**.

Publication bias was evaluated only for explosive power, as this was the sole outcome with more than ten effect sizes. Visual inspection of the funnel plot did not reveal obvious asymmetry (Figure 8). Consistently, Egger’s regression test showed no evidence of small-study effects (bias coefficient 2.90, 95% CI: −6.13 to 11.93; *p* = 0.49), indicating that publication bias is unlikely to have substantially influenced the pooled estimate for explosive power. Nevertheless, the overall number of available trials remains limited, and the results should be interpreted with appropriate caution.

### 3.5 Muscle damage biomarkers meta-analysis

#### 3.5.1 Effects of melatonin on muscle damage biomarkers

All effect sizes for muscle damage biomarkers are presented such that positive SMDs indicate protective effects (lower biomarker concentrations in the melatonin group), consistent with the direction coding described in the Methods section 2.5.

**Figure 9:**
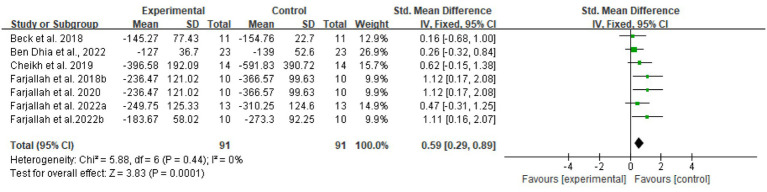
Forest plot of melatonin supplementation on CK.

**Figure 10:**
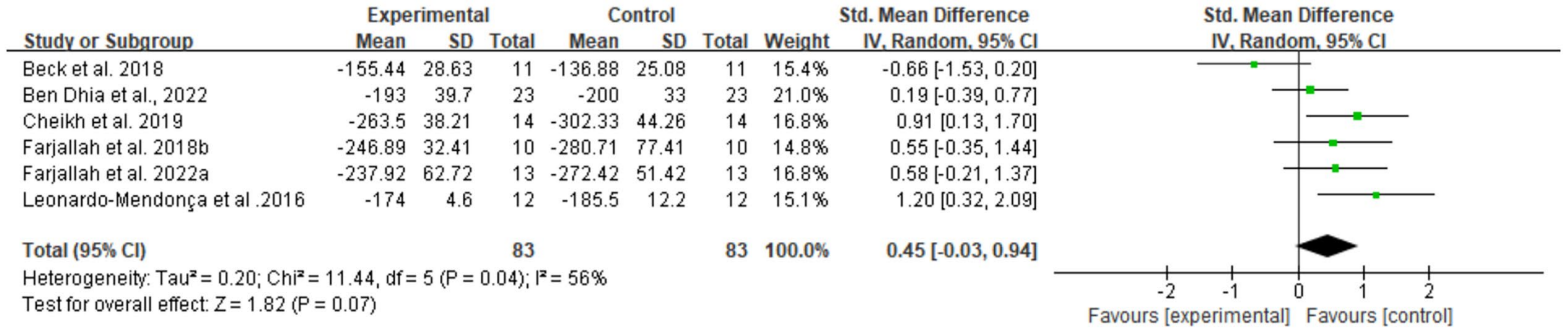
Forest plot of melatonin supplementation on LDH.

**Figure 11:**
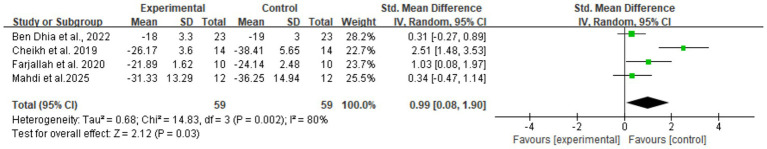
Forest plot of melatonin supplementation on ASAT.

Three biomarker-specific meta-analyses were performed for creatine kinase (CK), lactate dehydrogenase (LDH), and aspartate aminotransferase (ASAT) (Figures 9–11).

Across seven comparisons (*n* = 91), melatonin supplementation showed a pooled SMD of 0.59 (95% CI: 0.29–0.89, *p* < 0.0001, *I*^2^ = 0%) for CK under a fixed-effect model.

For LDH, six trials (*n* = 83) showed SMD 0.45 (95% CI: −0.03 to 0.94, *p* = 0.07, *I*^2^ = 56%) using a random-effects model.

For ASAT, four comparisons (*n* = 59) demonstrated SMD 0.99 (95% CI: 0.08–1.90, *p* = 0.03, *I*^2^ = 80%) using a random-effects model.

#### 3.5.2 Sensitivity and subgroup analyses

Sensitivity analyses using a leave-one-out approach for LDH and ASAT are presented in Supplementary Figure S2. Omission of any single study did not alter the pooled effect estimates for LDH. For ASAT, high heterogeneity (*I*^2^ = 80%) was primarily driven by one study, though removal did not change the direction of effect. Sensitivity analyses were not conducted for CK due to *I*^2^ = 0%.

For LDH, administration-to-exercise intervals of 2–6 h produced SMD 0.89 (95% CI: 0.40–1.38, *p* = 0.0004, *I*^2^ = 0%), whereas intervals ≤2 h showed SMD 0.10 (95% CI: −0.31 to 0.51, *p* = 0.62, *I*^2^ = 56%). Athlete populations demonstrated SMD 0.81 (95% CI: 0.39–1.22, *p* = 0.0001, *I*^2^ = 0%) compared to non-athletes SMD −0.08 (95% CI: −0.56 to 0.40, *p* = 0.76, *I*^2^ = 61%). Adolescents (≤18 years) showed SMD 0.69 (95% CI: 0.22–1.16, *p* = 0.004, *I*^2^ = 0%), whereas adults showed SMD 0.22 (95% CI: −0.21 to 0.64, *p* = 0.31, *I*^2^ = 77%) (see Table 4).

**Table 4:** The subgroup analysis results of LDH.

Study or subgroup	*n*	SMD	95% CI	*I*^2^ (%)	*p*
Population					
Athlete	4	0.81	[0.39, 1.22]	0	0.0001
No-athlete	2	−0.08	[−0.56, 0.40]	61	0.76
Age					
≤18	3	0.69	[0.22, 1.16]	0	0.004
>18	3	0.22	[−0.21, 0.64]	77	0.31
Time of melatonin administration					
Daytime	2	0.33	[−0.14, 0.79]	0	0.17
Evening	4	0.5	[−0.30, 1.30]	71	0.22
Administration-to-exercise interval					
≤2 h	3	0.1	[−0.31, 0.51]	56	0.62
2–6 h	3	0.89	[0.40, 1.38]	0	0.0004
Dosage					
≤5 mg	2	0.29	[−0.19, 0.78]	0	0.24
5–10 mg	3	0.29	[−0.62, 1.21]	74	0.53

SMD, standardized mean difference; CI, confidence interval.

#### 3.5.3 Publication bias

**Figure 12:**
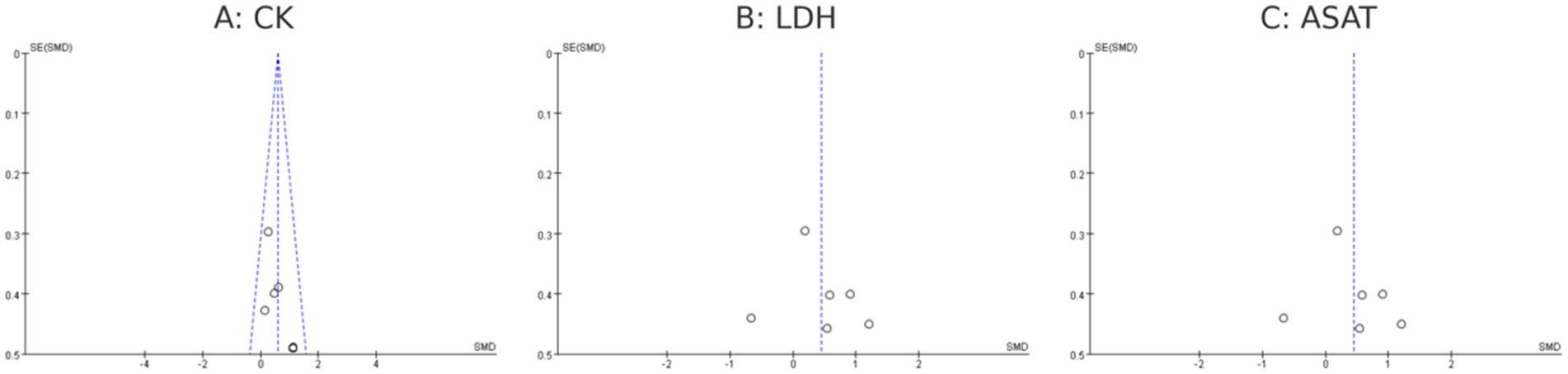
Funnel plot of melatonin supplementation on muscle damage biomarkers as **(A-C)**.

Publication bias was evaluated for CK, LDH, and ASAT as primary muscle damage biomarkers. Visual inspection of funnel plots revealed asymmetry for LDH and ASAT. For CK, while a slight asymmetry was observed, this should be interpreted cautiously given the small number of studies (*n* = 7) and absence of heterogeneity (*I*^2^ = 0%), with smaller studies reporting larger protective effects more frequently represented than those showing smaller or null effects. This pattern suggests potential publication bias may have influenced the pooled estimates for muscle damage biomarkers. The limited number of available studies (CK: *n* = 7, LDH: *n* = 6, ASAT: *n* = 4) warrants cautious interpretation of these findings (Figure 12).

### 3.6 GRADE recommendations

The strength of evidence according to GRADE criteria generated by the GRADEpro website^1^ is listed in Table 5.

**Table 5:** GRADE evidence profile for melatonin supplementation effects.

Certainty assessment							No. of patients		Effect	Certainty
No. of studies	Study design	Risk of bias	Inconsistency	Indirectness	Imprecision	Other considerations	Melatonin	Placebo	Absolute(95% CI)	
Explosive power										
12	Randomized trials	Not serious	Not serious	Not serious	Not serious	None	138	138	SMD 0.29 higher (0.05 higher to 0.53 higher)	⨁⨁⨁⨁
										High
Endurance performance										
8	Randomized trials	Not serious	Not serious	Not serious	Not serious	None	101	101	SMD 0.58 higher (0.29 higher to 0.87 higher)	⨁⨁⨁⨁
										High
Speed performance										
8	Randomized trials	Serious	Not serious	Not serious	Not serious	None	95	95	SMD 0.1 higher (0.4 lower to 0.6 higher)	⨁⨁⨁◯
										Moderate
Muscular strength										
5	Randomized trials	Not serious	Not serious	Serious	Serious	None	56	56	SMD 0.18 higher (0.2 lower to 0.55 higher)	⨁⨁◯◯
										Low
CK										
7	Randomized trials	Not serious	Not serious	Not serious	Not serious	None	91	91	SMD 0.59 higher (0.29 higher to 0.89 higher)	⨁⨁⨁⨁
										High
LDH										
6	Randomized trials	Not serious	Not serious	Not serious	Serious	None	83	83	SMD 0.45 higher (0.03 lower to 0.94 higher)	⨁⨁⨁◯
										Moderate
ASAT										
4	Randomized trials	Serious	Not serious	Not serious	Serious	Publication bias is strongly suspected	59	59	SMD 0.99 higher (0.08 higher to 1.9 higher)	⨁◯◯◯
										Very low

CI, confidence interval.

According to the results of the meta-analysis, a high GRADE recommendation supports the effects of melatonin supplementation in significantly improving explosive power and endurance performance in athletes compared with placebo. Additionally, High-certainty GRADE evidence supports melatonin’s protective effect in reducing creatine kinase levels (SMD = 0.59, *I*^2^ = 0%). Despite visual asymmetry in the funnel plot, the absence of heterogeneity and consistent effect across all studies suggests genuine biological effects rather than publication bias. However, the overall quality of the GRADE recommendations on the effect of melatonin supplementation in improving speed performance and reducing lactate dehydrogenase levels in athletes compared with placebo was considered low, with evidence characterized by substantial heterogeneity and imprecision. For muscular strength outcomes, the GRADE recommendation was also rated as low quality, suggesting that melatonin has no significant effect on maximal strength performance compared with placebo, according to the meta-analysis results. For aspartate aminotransferase, the GRADE recommendation was rated as very low quality, with evidence downgraded due to very serious inconsistency, serious imprecision, and small sample size, indicating that, despite statistical significance, confidence in this protective effect remains very limited.

## 4 Discussion

This systematic review and meta-analysis demonstrates that melatonin supplementation enhances athletic performance and reduces muscle damage through timing-dependent mechanisms, though evidence quality varies substantially across outcomes. High-certainty evidence supports benefits for endurance performance and creatine kinase reduction, while effects on explosive power are small and uncertain, and benefits for strength, speed, and coordination remain inconclusive.

The most critical finding is the timing-dependency of melatonin’s effects. Evening administration with >6-h intervals to exercise produced meaningful benefits, whereas daytime administration or shorter intervals showed minimal effects. This pattern reflects melatonin’s dual mechanisms: immediate sedative effects that may impair acute performance when administered shortly before exercise, and delayed restorative effects through sleep enhancement and circadian optimization that improve next-day performance. Multi-day supplementation demonstrated substantially larger effects than acute administration, indicating cumulative protective mechanisms operating through improved recovery rather than acute ergogenic action.

Our subgroup analyses revealed clear timing-dependent patterns: evening administration (SMD 0.45 for explosive power; SMD 0.73 for endurance) showed numerically larger effects than daytime administration (SMD 0.07 for explosive power; SMD 0.33 for endurance), though formal between-group comparisons were not conducted. More importantly, administration-to-exercise intervals exceeding 6 h produced moderate effects, whereas shorter intervals showed no effect. These timing-dependent effects likely reflect melatonin’s dual mechanisms: immediate sedative effects that may impair acute performance when administered shortly before exercise (16), and delayed restorative effects through sleep quality enhancement and circadian optimization that improve next-day performance (18, 20). The critical six-hour threshold suggests that melatonin’s ergogenic benefits manifest through overnight recovery processes rather than acute antioxidant protection during exercise itself. Multi-day supplementation protocols demonstrated substantially larger effects compared to acute administration, indicating cumulative protective mechanisms. Subgroup analyses further revealed that athlete populations demonstrated significantly greater muscle damage protection than non-athletes, suggesting that training status modulates melatonin’s protective efficacy.

When interpreting these timing-dependent effects, it is important to acknowledge that different administration protocols are likely to engage different underlying mechanisms. Acute daytime or pre-exercise dosing is more likely to influence arousal, thermoregulation, and acute antioxidant availability, and may even impair neuromuscular performance through sedative effects. In contrast, evening or pre-sleep multi-day protocols, especially when exercise is performed on the following day, are more plausibly acting through improvements in sleep and circadian alignment, together with cumulative antioxidant and anti-inflammatory adaptations.

### 4.1 Ecological validity: time-to-exhaustion vs. time-trial paradigms

Stratification by test modality revealed critical distinctions: melatonin significantly improved time-to-exhaustion performance (TTE: SMD 0.62, *p* = 0.008, *I*^2^ = 17%) but not time-trial performance (TT: SMD 0.38, *p* = 0.4, *I*^2^ = 65%). TTE protocols impose fixed intensity until exhaustion, reflecting tolerance to oxidative stress—directly targeted by melatonin’s antioxidant mechanisms. TT protocols require self-paced effort optimization, a cognitive task potentially impaired by melatonin’s sedative properties. The low heterogeneity in TTE (*I*^2^ = 17%) versus high heterogeneity in TT (*I*^2^ = 65%) further supports this distinction. These findings suggest melatonin primarily enhances training tolerance rather than competitive performance, as TTE improvements reflect the capacity to sustain high-intensity training stimuli while TT results indicate limited race-day ergogenic value. Melatonin appears most beneficial as a recovery aid during intensive training blocks rather than for acute performance enhancement.

Performance domain-specific analyses revealed that endurance performance showed moderate effects with high-certainty evidence, significantly exceeding other performance types. Acute melatonin administration substantially increased time to exhaustion at maximal aerobic capacity (6), while nocturnal melatonin enhanced next-day repeated sprint performance by reducing fatigue index and lowering peak heart rate (9). Notably, these benefits occurred independently of sleep parameter changes, suggesting direct ergogenic mechanisms rather than sleep-mediated effects alone. In contrast, muscular strength, neuromotor coordination, and speed performance demonstrated no significant benefits, though evidence certainty was low. The limited efficacy of strength-based activities aligns with observations that high-dose pre-exercise administration impaired anaerobic test performance through sedative effects (16), whereas nocturnal administration improved next-day strength and power output (18). Melatonin’s protective mechanisms operate through comprehensive antioxidant and anti-inflammatory pathways, significantly attenuating exercise-induced oxidative stress across multiple markers, including MDA reduction and enhanced antioxidant enzyme activities following both acute exhaustive exercise and multi-day intensive training (12–14, 19). Supplementation effectively attenuated post-exercise inflammatory responses through NF-κB inhibition, preventing excessive inflammatory cascades (11, 14, 43, 44).

Importantly, the high certainty of CK findings (*I*^2^ = 0% across 7 studies) contrasts with the moderate-to-very-low certainty for other biomarkers, suggesting that melatonin’s protective effect on muscle membrane integrity is robust and reproducible, while effects on other damage markers require further investigation.

Melatonin demonstrated multi-organ protective effects extending beyond skeletal muscle. Supplementation significantly decreased creatinine levels, indicating improved renal function, without affecting glucose, triglycerides, liver enzymes, or hematological parameters. Critically, melatonin demonstrated excellent safety across multiple physiological systems even at high doses (100 mg day^−1^) administered for 4 weeks, producing no adverse effects on hepatic enzymes, renal function markers, or hematological parameters (19). Across the included studies, melatonin doses ranging from 3 to 10 mg administered for durations up to 4 weeks consistently showed no clinically significant adverse effects on organ function (19). Melatonin’s tolerability is further confirmed by evidence demonstrating excellent safety even at high doses for extended periods. Additionally, melatonin enhanced aerobic performance while lowering total serum cholesterol (6), suggesting preferential lipid metabolism without adverse metabolic effects. However, timing-dependent considerations are crucial for safety. While most studies reported no adverse events, several documented melatonin-induced drowsiness or reduced alertness with daytime administration (16, 17, 21), emphasizing that timing is critical to avoid performance-impairing sedative effects.

### 4.2 Practical implications

Based on the evidence synthesis, nocturnal administration taken before sleep for several days during training camps or competition phases appears most effective for endurance athletes, supported by high-certainty evidence showing moderate performance improvements (27, 29, 30, 35). For explosive power activities, the benefits are small and of uncertain clinical significance (SMD 0.29). Athletes and practitioners should carefully weigh whether this small effect justifies supplementation. For strength, power, and speed activities, current evidence does not support melatonin supplementation, as no significant benefits were observed. For endurance performance enhancement, evening administration is recommended with exercise conducted the following day; daytime use within 6 h of exercise should be avoided. For individuals with overweight or obesity, lower doses administered pre-exercise provide substantial protection (7), though blood glucose monitoring is advisable. Critically, high-dose acute administration shortly before exercise should be avoided when athletes have adequate sleep, as sedative effects may impair immediate performance (16). Melatonin is generally well-tolerated, with doses up to 100 mg day^−1^ for 4 weeks producing no adverse effects on hepatic, renal, or hematological function (19). The optimal protocol comprises evening administration, multi-day supplementation during intensive training periods, and administration-to-exercise intervals exceeding 6 h, operating through dual pathways: direct antioxidant protection and sleep quality enhancement, facilitating recovery (47–48).

Moreover, most included trials did not characterize or stratify participants according to baseline endogenous melatonin concentrations. It therefore remains unclear whether the ergogenic and protective effects of melatonin supplementation differ between individuals with naturally low versus normal melatonin levels, which should be addressed in future studies.

### 4.3 Limitations

This meta-analysis has several limitations that warrant consideration. Small sample sizes across included trials limit statistical power and precision of effect estimates. Short-term evaluation periods preclude assessment of whether acute protective effects translate to enhanced chronic training adaptations. The predominance of male participants restricts generalizability to female athletes, and sex-specific responses remain uncharacterized. Publication bias concerns were identified for some muscle damage biomarkers (LDH and ASAT) based on funnel plot asymmetry and high heterogeneity, necessitating cautious interpretation of these specific outcomes. However, creatine kinase demonstrated high certainty with zero heterogeneity across studies, suggesting a robust and reproducible protective effect. The small number of studies (*n* = 7 for CK, *n* = 6 for LDH, *n* = 4 for ASAT) limits the statistical power of publication bias assessments through funnel plots alone. Heterogeneous outcome measures and exercise protocols across studies introduce substantial methodological variability. Future research should prioritize head-to-head comparisons of nocturnal versus acute administration protocols in well-powered trials, dose–response relationships across populations with varying training status, concurrent measurement of biochemical and performance outcomes at multiple time points, investigation of sex-specific responses and hormonal interactions in female athletes, longitudinal studies examining chronic training adaptations, and large-scale pre-registered trials to address publication bias and establish definitive efficacy and safety profiles across diverse athletic populations.

In addition, although several included trials administered melatonin in the evening or before bedtime, sleep-related outcomes were not consistently assessed. Therefore, we cannot determine whether the observed improvements in performance and attenuation of exercise-induced muscle damage were mediated by changes in sleep quality or duration. Future trials should systematically include objective and validated subjective sleep measures to clarify the extent to which melatonin’s effects are sleep-dependent.

Our categorical subgroup approach, while appropriate given sample size constraints, has inherent limitations, including increased Type I error risk from multiple comparisons. We minimized this risk through pre-specification of subgroups and conservative interpretation, focusing on consistency of effect direction. Meta-regression with continuous moderators would be preferable with larger sample sizes (≥40–50 studies), which future updated reviews should consider as more evidence accumulates.

## 5 Conclusion

This systematic review and meta-analysis provides high-certainty evidence that melatonin supplementation enhances endurance performance (moderate effect, SMD 0.58) training tolerance (TTE: SMD 0.62, *p* = 0.008) but not time-trial performance (TT: *p* = 0.4), positioning melatonin as a training recovery aid rather than competitive ergogenic aid, whereas evidence for explosive power shows only small benefits (SMD 0.29) of uncertain clinical significance while reducing exercise-induced muscle damage through timing-dependent mechanisms. Evening administration with administration-to-exercise intervals exceeding 6 h produced optimal benefits, while daytime administration showed negligible effects. Multi-day supplementation protocols yielded substantially greater efficacy than acute dosing, indicating cumulative protective mechanisms. Endurance performance demonstrated the most pronounced improvements with high-certainty evidence, whereas effects on explosive power were modest, and benefits for strength, coordination, and speed remain uncertain. For muscle damage protection, high-certainty evidence supports creatine kinase reduction (SMD = 0.59, 95% CI: 0.29–0.89, *I*^2^ = 0%). In contrast, LDH effects were non-significant (*p* = 0.07) with moderate-certainty evidence, and ASAT evidence is of very low quality. Publication bias concerns identified through funnel plot asymmetry primarily affect LDH and ASAT interpretations.

However, significant limitations constrain clinical translation. Variable evidence quality across performance domains, with high certainty for endurance and CK but low certainty for strength and speed outcomes, publication bias concerns for some biomarkers (LDH, ASAT), substantial publication bias affecting muscle damage outcomes, small sample sizes, short-term evaluation periods, and predominance of male participants, necessitate cautious interpretation. Future research should prioritize pre-registered, adequately powered trials examining long-term training adaptations, sex-specific responses, and mechanistic pathways underlying the dissociation between biochemical protection and functional outcomes. Until such evidence emerges, practitioners should apply melatonin supplementation primarily for endurance athletes during intensive training periods, while recognizing small and clinically uncertain benefits for explosive power, and lack of evidence supporting use for strength, power, and speed performance domains.

## Data Availability

The original contributions presented in the study are included in the article/Supplementary material, further inquiries can be directed to the corresponding author.

## Footnotes

1
https://www.gradepro.org/
