# Supplementary material for: Timing-dependent effects of melatonin supplementation on exercise performance and exercise-induced muscle damage: a systematic review and meta-analysis
Source: Front Nutr. 2026 Feb 13;13:1742464. doi: 10.3389/fnut.2026.1742464 (PMC12946080; doi:10.3389/fnut.2026.1742464)
Supplement: Supplementary file 1 [file Data_Sheet_1.pdf]

Table S1. The search strategy used for each database

| Databases. | Keywords | Results |
|---|---|---|
| Pubmed | ("Melatonin"[Mesh] OR melatonin[Title/Abstract]) AND (exercise[Title/Abstract] OR "physical activity"[Title/Abstract] OR sport*[Title/Abstract] OR athletic*[Title/Abstract]) AND (performance[Title/Abstract] OR "muscle damage"[Title/Abstract] OR biomarker*[Title/Abstract] OR CK[Title/Abstract] OR "creatine kinase"[Title/Abstract] OR LDH[Title/Abstract] OR "lactate dehydrogenase"[Title/Abstract] OR MDA[Title/Abstract] OR malondialdehyde[Title/Abstract]) | 154 |
| Web of science | TS=(melatonin OR "melatonin supplementation" OR "melatonin intake")<br>AND TS=(exercise OR "physical activity" OR sport* OR athletic* OR training OR workout)<br>AND TS=(performance OR endurance OR recovery OR fatigue OR "muscle damage" OR "oxidative stress" OR inflammation OR biomarker* OR CK OR "creatine kinase" OR LDH OR "lactate dehydrogenase" OR MDA OR malondialdehyde OR antioxidant) | 42 |
| SPORTDiscus (EBSCO) | (TI OR AB OR SU)(melatonin OR "melatonin supplementation" OR "melatonin intake")<br>AND<br>(TI OR AB OR SU)(exercise OR "physical activity" OR sport* OR athletic* OR training OR workout)<br>AND<br>(TI OR AB OR SU)(performance OR endurance OR recovery OR fatigue OR "muscle damage" OR "oxidative stress" OR inflammation OR biomarker* OR CK OR "creatine kinase" OR LDH OR "lactate dehydrogenase" OR MDA OR malondialdehyde OR antioxidant)<br>Filters: English language; Peer-reviewed journals; Humans. | 16 |
| Cochrane Library | :(melatonin OR "N-acetyl-5-methoxytryptamine" OR melatonergic OR "pineal hormone") AND (sport* OR exercise OR athlete* OR training OR "physical activity" OR "physical exercise" OR fitness OR "physical conditioning" OR workout*) AND (performance OR "athletic performance" OR "exercise performance" OR "physical performance" OR endurance OR "aerobic capacity" OR "anaerobic capacity" OR strength OR "muscular strength" OR power OR "muscular power" OR speed OR agility OR "exercise capacity" OR VO2max OR "maximal oxygen consumption" OR "oxygen uptake" OR "time to exhaustion" OR "time trial" OR sprint* OR jump* OR "reaction time" OR "work capacity" OR "peak power" OR stamina) | 204 |

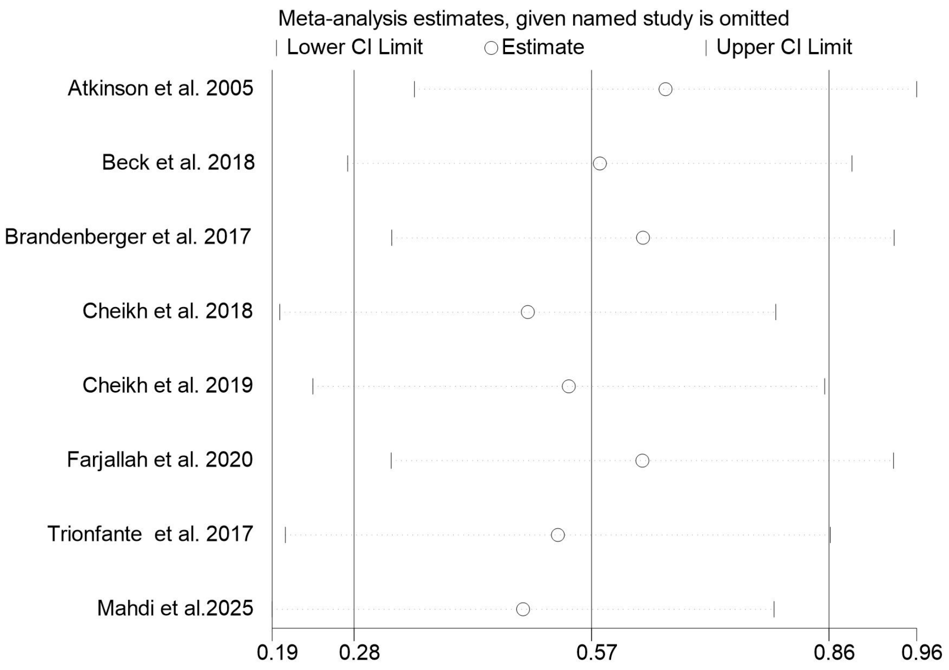


Endurance Performance

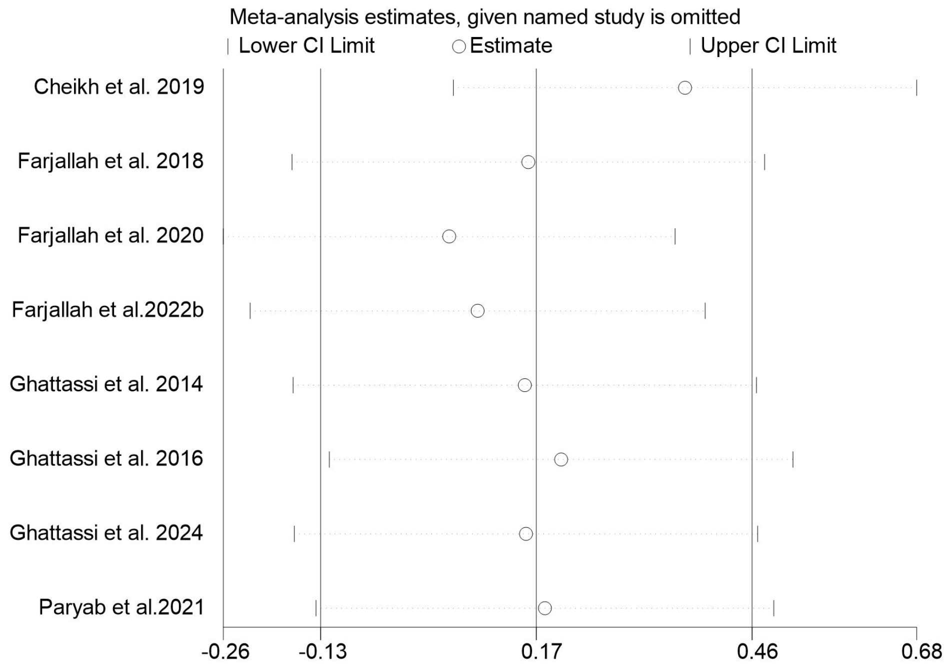


Speed Performance

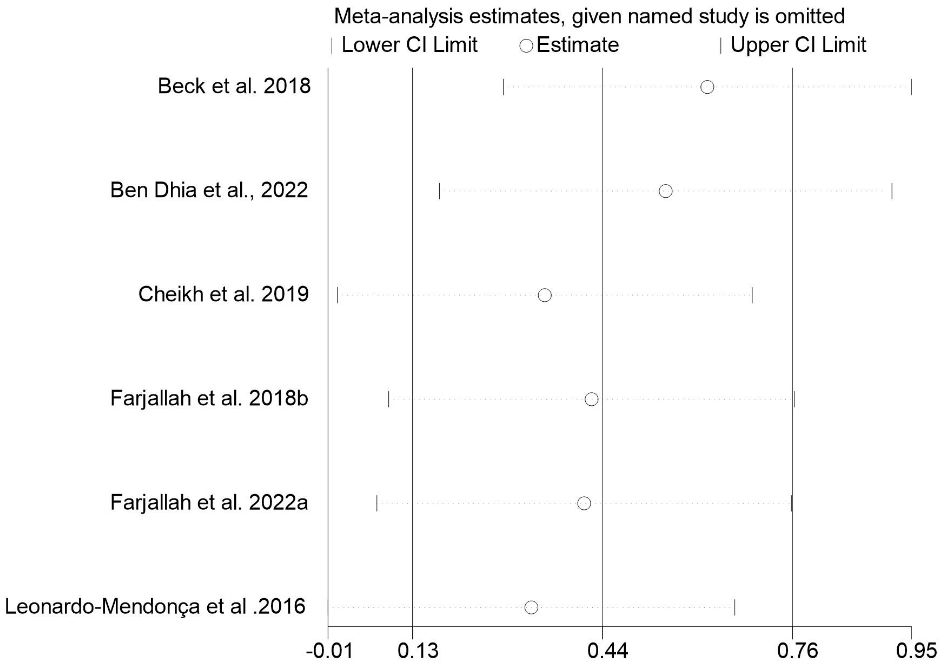

Meta-analysis estimates, given named study is omitted
Lower CI Limit
Estimate
Upper CI Limit
Beck et al. 2018
Ben Dhia et al., 2022
Cheikh et al. 2019
Farjallah et al. 2018b
Farjallah et al. 2022a
Leonardo-Mendonça et al .2016
-0.01
0.13
0.44
0.76
0.95


LDH

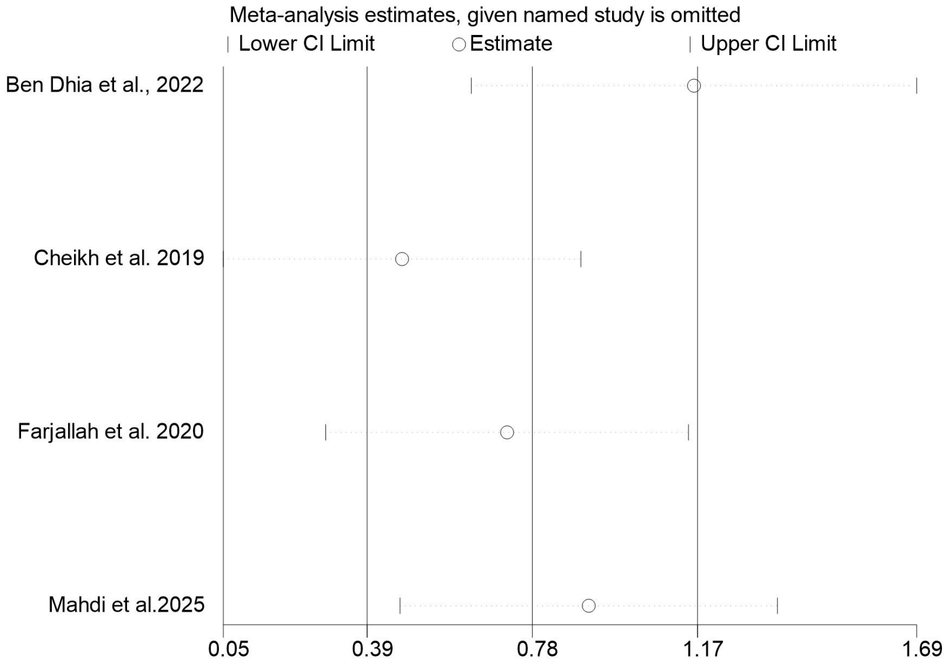

Meta-analysis estimates, given named study is omitted
Lower CI Limit
Estimate
Upper CI Limit
Ben Dhia et al., 2022
Cheikh et al. 2019
Farjallah et al. 2020
Mahdi et al.2025
0.05
0.39
0.78
1.17
1.69


ASAT
